# Mitotane Concentrations Influence Outcome in Patients with Advanced Adrenocortical Carcinoma

**DOI:** 10.3390/cancers12030740

**Published:** 2020-03-20

**Authors:** Soraya Puglisi, Anna Calabrese, Vittoria Basile, Filippo Ceccato, Carla Scaroni, Barbara Altieri, Silvia Della Casa, Paola Loli, Rosario Pivonello, Maria Cristina De Martino, Letizia Canu, Marco Russo, Giuseppe Badalamenti, Massimo Torlontano, Antonio Stigliano, Francesco Ferraù, Giorgio Arnaldi, Laura Saba, Alessandra Quirino, Paola Perotti, Paola Berchialla, Massimo Terzolo

**Affiliations:** 1 Internal Medicine, Department of Clinical and Biological Sciences, San Luigi Gonzaga Hospital, University of Turin, Orbassano, 10043 Turin, Italy; sorayapuglisi@yahoo.it (S.P.); basile_vittoria@libero.it (V.B.); laura.saba@unito.it (L.S.); alessandraquirino1994@gmail.com (A.Q.); oncotrial.sanluigi@gmail.com (P.P.); massimo.terzolo@unito.it (M.T.); 2 Endocrinology Unit, Department of Medicine DIMED, University-Hospital of Padua, 35128 Padua, Italy; ceccato.filippo@gmail.com (F.C.); carla.scaroni@unipd.it (C.S.); 3 Division of Endocrinology and Metabolic Diseases, University-Hospital Gemelli, IRCSS, Catholic University of the Sacred Heart, 00168 Rome, Italy; altieri.barbara@gmail.com (B.A.); silvia.dellacasa@unicatt.it (S.D.C.); 4 Endocrinology, Hospital Niguarda Ca’ Granda, 20121 Milan, Italy; paola.loli@clinicasancarlo.it; 5 Dipartimento di Medicina Clinica e Chirurgia, Sezione di Endocrinologia, Università Federico II di Napoli, 80131 Naples, Italy; rosario.pivonello@unina.it (R.P.); demartino.mc@gmail.com (M.C.D.M.); 6 Endocrinology Unit, Department of Experimental and Clinical Biomedical Sciences, University of Florence, 50134 Florence, Italy; letizia.canu@unifi.it; 7 Endocrinology Division, Department of Clinical and Experimental Medicine, ARNAS Garibaldi, University of Catania, 95122 Catania, Italy; mruss@hotmail.it; 8 Department of Surgical, Oncological, and Oral Sciences, Section of Medical Oncology, University of Palermo, 90127 Palermo, Italy; giuseppe.badalamenti@unipa.it; 9 Unit of Endocrinology, Fondazione IRCCS Casa Sollievo della Sofferenza, 71013 San Giovanni Rotondo, Italy; m.torlontano@operapadrepio.it; 10 Endocrinology, Department of Clinical and Molecular Medicine, Sant’Andrea Hospital, Sapienza University of Rome, 00189 Rome, Italy; antonio.stigliano@uniroma1.it; 11 Department of Human Pathology of Adulthood and Childhood ‘G. Barresi’, University of Messina, 98125 Messina, Italy; francesco.ferrau1@gmail.com; 12 Division of Endocrinology, Department of Clinical and Molecular Sciences (DISCLIMO), Polytechnic University of Marche, 60121 Ancona, Italy; gioarnaldi@gmail.com; 13 Statistical Unit, Department of Clinical and Biological Sciences, Orbassano, University of Turin, 10143 Orbassano, Italy; paola.berchialla@unito.it

**Keywords:** adrenal cancer, mitotane, prognosis, recurrence, survival

## Abstract

Mitotane is the main option of treatment for advanced adrenocortical carcinoma (ACC). However, limited evidence is available regarding the impact of plasma mitotane levels on patient outcome. To address this question, we retrospectively analyzed patients with advanced ACC treated with mitotane for ≥3 months, with ≥3 measurements of plasma mitotane reported in the Lysosafe Online^®^ database (HRA Pharma, France), followed at 12 tertiary centers in Italy from 2005 to 2017. We identified 80 patients, initially treated with mitotane alone (56.2%) or plus chemotherapy (43.8%). The preference toward combination therapy was given to de novo stage IV ACC and younger patients. After the first line of treatment, 25% of valid cases experienced clinical benefit (14.5% objective response, 10.5% stabilization of disease) and 75% progression, without differences between the groups of treatment. Patients with progression had a lower time in the target range (TTR) of plasma mitotane and an unfavorable outcome. Death occurred in 76.2% of cases and multivariate analysis showed that clinical benefit after first treatment and longer TTR were favorable predictors of overall survival (OS). In conclusion, the present findings support the importance of mitotane monitoring and strengthen the concept of a therapeutic window for mitotane.

## 1. Introduction

The last years have witnessed an unprecedented development in medical oncology, with the introduction in clinics of targeted drugs and immune therapies that changed the prognosis of several cancers. This advancement has not included medical therapy of adrenocortical carcinoma (ACC), which remains based on a drug that has been developed in the sixties, mitotane. 

Mitotane is approved by US and European regulatory agencies for treatment of advanced ACC, albeit being increasingly used in the adjuvant setting also [1]. The European Society of Endocrinology and the European Network for the Study of Adrenal Tumors (ESE-ENSAT) guidelines on management of ACC suggest mitotane monotherapy for patients harboring advanced ACC with favorable prognostic parameters, while mitotane associated with chemotherapy is indicated in patients with perceived worse prognosis (i.e., aggressive tumors) [2]. This indication has a limited evidence base, since only one randomized controlled study is available on the association of mitotane with chemotherapy in advanced ACC [3].

Therefore, we thought it of interest to do a survey on the use of mitotane as treatment of advanced ACC not amenable to surgical resection in referral centers for ACC care in Italy. The study aim was to assess whether mitotane levels impact on the response to treatment and patient outcome. Studies have shown the therapeutic value of mitotane concentrations is 14–20 mg/L; however, the validation of this range was done using the peak mitotane level [4,5,6], which cannot give an adequate representation of a chronic exposure to mitotane, being a measurement at a single point in time, or the percentage of mitotane measurements in a range [7,8], which is strongly dependent on the number of available measurements. To get a better assessment of the exposure to mitotane over time, we assessed the time in target range (TTR), defined as the number of months in which mitotane concentrations were ≥14 mg/L. The TTR has been introduced for assessing the optimal exposure to warfarin therapy [9], and since there is an analogy between mitotane and warfarin concerning the need to get drug levels within a defined range of concentrations, we evaluated whether the TTR may influence patient survival.

## 2. Results

**Figure 1:**
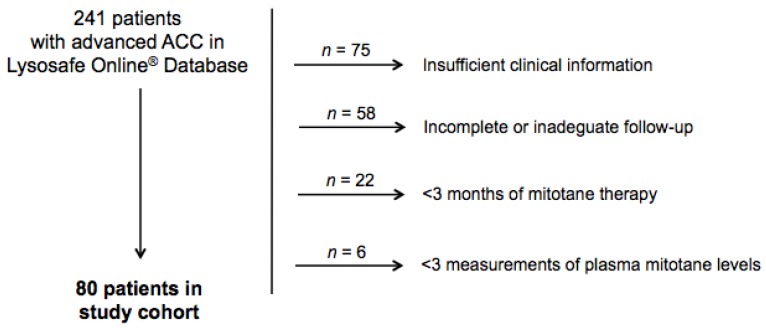
Study cohort.

From a total of 241 patients with advanced ACC on the Lysosafe^®^ Online database, 80 patients fulfilled inclusion/exclusion criteria and were retrospectively included in the study (Figure 1). Baseline characteristics of the patients are reported in Table 1.

The median follow-up was 33 (22–51.2) months. Twenty-four patients were previously treated with adjuvant mitotane before ACC recurrence while 56 patients started mitotane (±chemotherapy) as first-line medical treatment of ACC recurrence or advanced disease at diagnosis. Median duration of palliative treatment was 33 (22–49) months, with a median of 8 (5–12) measurements of plasma mitotane concentration and a median time interval between two consecutive measurements of 2 (1–3) months. At the end of the follow-up, 14 patients (17.5%) were still on mitotane therapy, after a median of 67 (43–102) months of palliative mitotane treatment, and 48 (72.7%) were treated until death, with a median duration of 31 (21–44) months. Other causes of treatment discontinuation were ACC progression (*n* = 10), unknown (*n* = 5), or patient’s decision (*n* = 3).

In the overall group, the achievement of target mitotane levels required a median time of 6 (3–9) months from the start of therapy while 14 patients (17.5%) never achieved levels ≥ 14 mg/L. The peak of plasma mitotane concentrations was 20.8 (14.8–25.0) mg/L, which was reached after a median of 11 (6–20) months.

**Figure 2:**
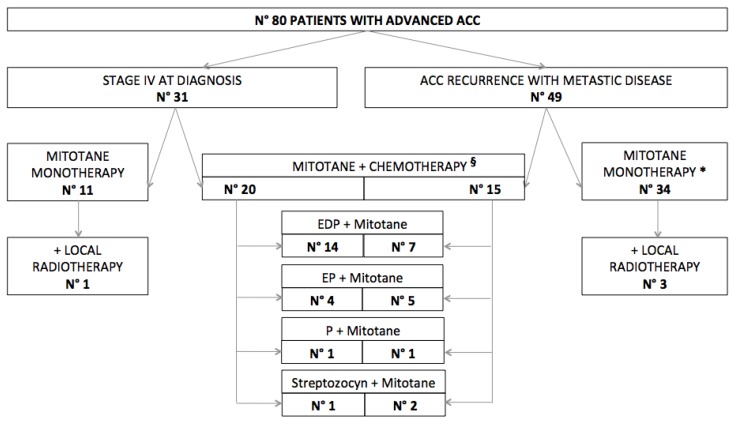
Management of advanced adrenocortical carcinoma (ACC)in our series. * 17 patients were previously on adjuvant mitotane. **^§^** 7 patients were previously on adjuvant mitotane. EDP = etoposide, doxorubicin and cisplatin. EP = etoposide and cisplatin. P = cisplatin.

Forty-five patients (56.2%) were initially treated only with mitotane (4 of which received concomitant local radiotherapy) while 35 (43.8%) were treated with a combination of chemotherapy and mitotane, in most cases with the EDP-M (etoposide, doxorubicin, and cisplatin, plus mitotane) regimen (Figure 2).

The comparison between the baseline characteristics of these two groups showed that patients treated with the combination of chemotherapy and mitotane were younger (43, 33–58 years, vs. 54, 45–62 years; *p* = 0.036) and with worse presentation at diagnosis (de novo ENSAT stage IV, 57.1% vs. 24.4%; *p* = 0.012) (Table 2). 

During the entire period of follow-up, 12 patients were treated only with mitotane while the remaining 68 received mitotane in combination with one or more chemotherapy treatments (in 61 cases, at least one regimen including platinum compound). 

The comparison between the baseline characteristics of these two groups showed that patients treated with multiple lines of treatment were younger than those treated with mitotane only (48.5, 35–58.2 years, vs. 58.5, 52.7–69.2 years; *p* = 0.023) (Table 3). We found that patients treated only with mitotane achieved a higher peak of mitotane concentrations (26.4, 21.7–29.2 mg/L, vs. 19.2, 14.7–24.6 mg/L, *p* = 0.028) and had a more favorable outcome (6/12 (50%) vs. 13/55 (23.6%) patients alive at last follow-up, *p* = 0.022).

Considering the first line of treatment, 19 patients (23.7%) experienced clinical benefit (10 out of 35 (28.6%) patients treated with mitotane + chemotherapy and 9 out of 45 (20%) patients in mitotane monotherapy), of whom 11 (57.9%) had an objective response (8 partial, 3 complete) and 8 (42.1%) had stabilization of disease, while the remaining 57 (71.2%) had progression (24 out of 35 (68.6%) patients treated with mitotane + chemotherapy and 33 out of 45 (73.3%) patients in mitotane monotherapy), according to the RECIST 1.1 criteria [10] (data not available for 4 patients). 

The comparison between the baseline characteristics of these two groups is given in Table 4. We found that patients with ACC progression had a lower TTR (8.6, 0.8–14.1 months, vs. 15.8, 2.7–32.7 months; *p* = 0.033) and an unfavorable outcome (7/57 patients (12.3%) vs. 12/19 (63.2%) patients alive at last follow-up, *p* = 0.022).

Considering the entire cohort of patients, death occurred in 61 cases (76.2%). Median overall survival (OS) was 35 months (CI95%, 31–49). Multivariate analysis showed that clinical benefit after the first-line treatment and the TTR were independent predictors of OS (Table 5).

## 3. Discussion

Management of patients with advanced ACC also remains a challenge in referral centers. ACC may present with metastatic disease at diagnosis in about one quarter of cases, or progress to advanced disease after an initial apparently complete resection [11,12]. The prognosis of advanced ACC is generally poor when surgery is unfeasible, with a reported 5-year survival less than 15% [11,13]. 

Our study shows that less than a quarter of patients were alive after 3 years of follow-up, with a median survival of 35 months. These data confirm the high mortality of advanced ACC [12,14]; however, they also follow the trend of a better prognosis observed in the most recent studies [13]. The present study shows that the lung is the organ most commonly involved by metastatic spread, with a higher frequency than previously reported in clinical series [14] but with a similar rate of autoptic [15] or surgical series [16,17]. The disease burden in our cohort was remarkable, with the presence of ≥two organs involved in about two thirds of cases, and also hormone secretion was found in a similar percentage of cases at diagnosis. It is worth noting that a remarkable number of secreting tumors at diagnosis recurred as not secreting tumors (secreting tumors 66.2% at diagnosis vs. 32.5% at the start of palliative treatment). The patient cohort enrolled in the FIRMACT study had quite a higher tumor burden and less secreting tumors, and this difference likely results from the specific inclusion criteria of that study (all patients were treated with chemotherapy in addition to mitotane) [3]. 

The present study reports on the current practice in the management of advanced ACC, either stage IV at diagnosis or recurrent ACC following initial surgery, focusing on the first-line treatment. It is generally agreed that the two major options are mitotane monotherapy or mitotane combined with chemotherapy, with a regimen including platinum compounds [12,14]. The choice depends on patient conditions, tumor characteristics, and center preference. The standard chemotherapy regimen is EDP-M, introduced by a multicenter prospective phase II study carried out in Italy [18,19] and validated in a worldwide prospective randomized phase III clinical trial [3]. Our series demonstrated that this finding has been implemented in current practice, since EDP was the regimen of choice as the first treatment in most of our patients. In some cases, the parent regimens EP, or cisplatin monotherapy, were used due to limited toxicity. Conversely, the combination of streptozotocyn and mitotane was employed in less than 10% of cases.

In our cohort, the two options (mitotane alone or plus chemotherapy) were almost equally chosen; however, preference toward mitotane combined with chemotherapy was given to de novo stage IV ACC and younger patients. This choice likely reflects the perception that a metastatic presentation at diagnosis implies an aggressive ACC and that younger patients are fit to sustain chemotherapy-related toxicity. However, mitotane remained the backbone of therapy because the duration of treatment was prolonged till patient death in most cases while different lines of treatment (cytotoxic drugs, loco-regional treatments) were superimposed during the disease course. The practice of continuing mitotane indefinitely, despite ACC progression, has been recently criticized [20], although we lack clear rules for mitotane discontinuation [2]. 

A small subset of our patients were treated with mitotane without any other additional systemic treatment. Interestingly, these patients had a more favorable outcome, and this likely represents a selection bias because more aggressive tumors usually undergo multiple lines of treatment. The inclusion of this patient cohort with less aggressive ACC is one of the factors that may explain the long OS observed in the present study. Not surprisingly, higher mitotane concentrations were attained in such patients since the combination with cytotoxic agents increases toxicity and makes it difficult to give high doses of mitotane. In a small prospective trial of 12 weeks, including 40 mitotane-naïve patients with metastatic ACC, assigned to a low- or high-dose mitotane regimen, the high-dose regimen resulted in higher exposure to mitotane in patients not receiving concomitant chemotherapy, despite cumulative doses not being significantly different among the subgroups [21]. 

A recent retrospective study including 127 patients with advanced ACC treated with mitotane monotherapy introduced the concept that either a low tumor burden (<10 tumor lesions) or longer recurrence-free survival (RFS) after primary surgery (≥360 days) are characteristics predicting treatment efficacy [6]. We did not find any difference on tumor burden, expressed as the number of metastatic organs, although we did not capture data on the number of metastatic lesions. However, RFS was almost double in the cohort of patients treated with mitotane alone, despite levels of significance not being reached for the low numbers, thus confirming the validity of the concept that tumors with lower proliferation capability (heralded by prolonged RFS) are best suited for mitotane monotherapy. 

An interesting finding of the present study is that the TTR of plasma mitotane concentrations is able to predict survival, with a higher value associated with longer survival. This was consistent with the previous literature, which established the therapeutic value of mitotane concentrations of 14–20 mg/L [4,5,6,7,8,22]. We adopted the TTR using a concept analogous to warfarin treatment [9] that we recently used in a study on adjuvant mitotane treatment [23]. In that study, we found that a higher TTR was associated with a reduced risk of ACC recurrence [23]. In the present study, we found that patients with ACC progression had a lower time in the target range than patients experiencing clinical benefit from first-line treatment, and in multivariable analysis, TTR was a predictor of OS. This confirms that TTR could represent a valuable measure of mitotane efficacy also in advanced ACC. 

In our study, the overall activity of first-line treatment was limited, with a low number of objective responses (13.7%) and of patients experiencing clinical benefit (23.7%), with no observed difference between patients treated with mitotane + chemotherapy or mitotane monotherapy. We observed that a lower number of patients with advanced ACC benefitted from first-line treatment, either mitotane + chemotherapy or mitotane monotherapy, compared to the FIRM-ACT study and two recent retrospective studies [3,6,22]. However, the present study was not specifically designed to analyze the efficacy of treatment. 

Strengths of the present study are the large data set of patients with available clinical information and data on mitotane measurement, considering the rarity of ACC and the high mortality in the setting of advanced disease. On the other hand, we are aware that our analysis is limited by its retrospective and multicenter nature. Therefore, we did not evaluate progression-free survival, which is heavily influenced by variable schedules of restaging in retrospective studies, and we only considered overall survival, taking into account that the inclusion criteria produced an immortal time of three months, and that we included patients with at least three mitotane measurements.

## 4. Materials and Methods 

For this study, we invited 13 tertiary centers for the care of ACC patients in Italy. Twelve centers accepted the invitation to participate in the survey, providing clinical, pathological, and biochemical data of all patients with advanced ACC who had been proactively followed at the center and treated with mitotane. We retrieved the data of patients who were treated from July 2005 to March 2017. Follow up for this study was closed on 1 November 2019. The institutional ethics committee of all centers approved the study, and all patients signed written informed consent (Ethical Committee of AOU San Luigi Gonzaga (Orbassano, Turin, Italy) and of AA.SS.LL. TO3–TO4–TO5 based at AOU San Luigi Gonzaga (Orbassano, Turin, Italy). Number of dossier 36/2012, validate on the session of 22 February 2012). 

Inclusion criteria of the study were as follows: Age ≥ 18 years, pathologically confirmed diagnosis of ACC, availability of computed tomography (CT) or magnetic resonance imaging (MRI) scans, complete follow-up information, treatment with mitotane (all patients received the same mitotane formulation, Lysodren^®^ 500 mg tablets) for ≥ 3 months, and with > 3 measurements of plasma mitotane concentrations reported on the Lysosafe Online^®^ database. Exclusion criteria were as follows: Incomplete tumor staging, history of other previous or concomitant malignancies, and incomplete follow-up information.

Patients’ charts were reviewed and the following information was retrieved for the study: Gender, date of birth, date of diagnosis, hormone secretion and tumor stage at diagnosis, pathology report, date of recurrence (in case of previous adjuvant treatment), date of start of palliative treatment, hormone secretion and ACC stage at start of palliative treatment, number and type of organs/systems with metastasis at start of palliative treatment, and last follow-up or death. Date of diagnosis was defined as the date of surgery or the date of biopsy for tumors not operated on. Biochemical confirmation of hormone excess was requested to categorize an ACC as hormone secreting. Tumor stage was established according to the ENSAT classification (I and II, confined tumor; III, positive lymph nodes or infiltrating neighboring organs/veins without distant metastases; IV, distant metastases) [24]. Date of recurrence was defined as the date of radiological evidence of a new lesion. A questionnaire was sent to the participating centers to retrieve the information requested for the study; moreover, centers were asked about indications, timing of initiation and discontinuation, reasons for discontinuation of mitotane treatment, and follow-up modality. Duration of treatment was calculated from the date of the initiation of mitotane therapy until the discontinuation of treatment, or the end of follow-up, whichever occurred first. Treatment response was evaluated according to routine radiologic assessment and qualified patients were classified on the basis of their best response to the first line of treatment using Response Evaluation Criteria in Solid Tumors (RECIST) 1.1 [10]: Complete response (CR), partial response (PR), stable disease (SD), and progressive disease (PD). We stratified patients in two groups: The first group includeed patients with clinical benefit (CR, PR, SD) vs. patients with progression (PD). 

For the analysis of plasma mitotane concentrations, we calculated the time in target range (TTR), defined as the number of months in which mitotane concentrations were ≥14 mg/L, a value considered as the lower limit of the target range [5,6,8], for all patients. Based on the concept of the “time in therapeutic range” used for monitoring warfarin therapy [9], we assumed that a linear relationship existed between consecutive values when a measurement was not available.

Mitotane concentrations were retrieved from the Lysosafe Online^®^ database, available at www.lysosafe.com. Lysosafe Online^®^ is a login-protected website that stores mitotane plasma concentrations of patients treated by physicians who have registered with the Lysosafe^®^ service, a free-of-charge service of the measurement of plasma mitotane concentrations in ACC patients offered by HRA Pharma to European prescribers since 2005 and associated with the use of Lysodren^®^. Samples were collected at the centers, sent to a centralized laboratory, extracted by precipitation with ethanol, and tested by a standardized gas chromatography/mass spectrometry method. Plasma mitotane values of any patient were available for the treating physician on www.lysosafe.com, in a historical and graphic plot that matches mitotane levels with the relative Lysodren^®^ dose. Patient data are anonymous during the whole process since patients are recorded using an acronym and their date of birth.

All communication concerning the study between centers was by email, and a meeting was organized to make the process of data capture more homogeneous.

### Statistical Analysis

Categorical data are presented as counts and percentages. Continuous data are presented as medians and interquartile ranges (IQR). Differences in categorical variables were analyzed by means of the chi-squared test or Fisher test as appropriate, while differences in continuous variables were analyzed by the Mann–Whitney U test. The survival curves were estimated with the Kaplan–Meyer product limit method. RFS was calculated from the time of initial surgery to the first radiological evidence of recurrence. OS was calculated from the start of palliative treatment to the date of death. Patients who did not experience either of those events (recurrence or death) were censored at the date of the last follow-up visit for the specific survival analysis. Cox proportional hazards regression models were fitted to determine prognostic factors on OS. The following potential predictive factors for OS were investigated: Patient sex, age, and hormone secretion at the starting of palliative treatment, length of RFS (0 for de novo stage IV), the number of organs with metastasis at the start of palliative treatment, the time elapsed to get the first plasma mitotane level at target during palliative treatment, the peak of mitotane concentrations during palliative treatment, and response to treatment.

Since variable selection based on univariate analysis cannot properly control for a potential spurious relationship [25], the best subset regression approach was chosen for building a multivariate model [26]. According to this approach, all the possible combinations of the candidate variables were considered, then model selection was based on the Akaike information criteria method [27].

All reported *p* values are two-sided. The *p* values less than 0.05 were considered as statistically significant. The statistical analyses were performed with Statistica (StatSoft) (Dell Software, Round Rock, TX, USA) and R version 3.5.1 (R Core Team, St. Louis, MO, USA).

## 5. Conclusions

In reference centers in Italy, the two options for treatment of advanced ACC (mitotane alone or plus chemotherapy) were almost equally chosen, but mitotane remained the backbone of therapy because the duration of treatment was prolonged till patient death in most cases. The observation that the TTR of plasma mitotane concentrations has prognostic implications is novel and supports the importance of mitotane monitoring and the value of the concept of a therapeutic window for mitotane.

## Figures and Tables

**Table 1:** Baseline features of patients.

Characteristics	Valid Cases (*N*)	Values
Gender, *N* (%)	80	
Male		25 (31.2%)
Female		55 (68.8%)
**Age at diagnosis**, year	80	
Median (IQR)		50 (36–59)
**Tumor stage at diagnosis**, *N* (%)	80	
Stage I		3 (3.7%)
Stage II		28 (35%)
Stage III		18 (22.5%)
Stage IV		31 (38.8%)
**Hormone secretion at diagnosis**, *N* (%)	77	
Yes		51 (66.2%)
No		26 (33.8%)
**Weiss score**	51	
Median (IQR)		6 (5–7)
**Ki67**	55	
Median (IQR)		25 (13–38)
≤10%		14 (25.5%)
>10%		41 (74.5%)
**Hormone secretion at start of palliative treatment**, *N* (%)	77	
Yes		25 (32.5%)
No		52 (67.5%)
**ENSAT tumor stage at start of palliative treatment**, *N* (%)	80	
Stage I		0 (0%)
Stage II		0 (0%)
Stage III		7 (8.7%)
Stage IV		73 (91.3%)
**Number of metastatic organs**	78	
Median (IQR)		2 (1–2)
1 organ, *N* (%)		29 *(37.2%)*
2 organs, *N* (%)		34 *(43.6%)*
3 organs, *N* (%)		10 *(12.8%)*
≥ 4 organs, *N* (%)		5 *(6.4%)*
**Organ/system involved**	78	
Lungs		47 *(60.3%)*
Liver		37 *(47.4%)*
Lymphatic system		15 *(49.2%)*
Local site (vena cava, adrenal loggia)		13 *(16.7%)*
Peritoneum and retroperitoneum		8 *(10.3%)*
Kidney		8 *(10.3%)*
Skeletal system		7 (9.0%)
Spleen		3 *(3.8%)*
Abdominal muscles (psoas, diaphragm)		3 *(3.8%)*
Colon		2 *(2.6%)*

IQR = interquartile range. N = number of patients.

**Table 2:** Comparison between patients starting palliative treatment with mitotane monotherapy vs. patients starting with the association mitotane + chemotherapy.

Characteristics	Mitotane Monotherapy N° 45	Mitotane + Chemotherapy N° 35	*p* Value
**Gender, *(valid cases)***	***(45)***	***(35)***	0.43
Male, *N* (%)	12 *(26.7)*	13 *(37.1)*	
Female, *N* (%)	33 *(73.3)*	22 *(62.9)*	
**Age at time of palliative treatment**, year ***(valid cases)***			
		
	***(45)***	***(35)***	**0.036**
Median (IQR)	54 (45–62)	43 (33–58)	
**Hormone secretion at start of palliative treatment** ***(valid cases)***			
		
	***(42)***	***(31)***	0.085
Yes, *N* (%)	9 *(21.4)*	14 *(45.2)*	
No, *N* (%)	33 *(78.6)*	17 *(54.8)*	
**Number of metastatic organs** ***(valid cases)***			
		
	***(43)***	***(35)***	0.92
≤ 2 organs, *N* (%)	35 *(81.4)*	28 *(80)*	
> 2 organs, *N* (%)	8 *(18.6)*	7 *(20)*	
*De novo* stage IV * ***(valid cases)***			
	***(45)***	***(35)***	**0.012**
*N* (%)	11 (24.4)	20 (57.1)	
**Previous adjuvant therapy** ***(valid cases)***			
	***(45)***	***(35)***	0.17
Yes, *N* (%)	17 *(37.8)*	7 *(20)*	
No, *N* (%)	28 *(62.2)*	28 *(80)*	
**Previous RFS** ***(valid cases)***			
	***(18)***	***(7)***	0.74
Median (IQR)	16 (6–26)	16 (5–54)	

* De novo stage IV means that patients were diagnosed with stage IV ACC. IQR = interquartile range. N = number of patients. RFS = recurrence free survival. Statistically significant outcomes are presented in bold.

**Table 3:** Comparison between patients treated with mitotane monotherapy during all follow-up vs. patients treated with different lines of treatment.

Characteristics	Mitotane Monotherapy N° 12	Mitotane + Chemotherapy N° 68	*p* Value
**Gender, *(valid cases)***	***(12)***	***(68)***	0.90
Male, *N* (%)	4 *(33.3%)*	21 *(30.9%)*	
Female, *N* (%)	8 *(66.6%)*	47 *(69.1%)*	
**Age at time of palliative treatment**, year ***(valid cases)***			
		
	***(12)***	***(68)***	**0.023**
Median (IQR)	58.5 (52.7–69.2)	48.5 (35–58.2)	
**Hormone secretion at start of palliative treatment** ***(valid cases)***			
		
	***(11)***	***(62)***	0.80
Yes, *N* (%)	3 *(27.3)*	20 *(32.3)*	
No, *N* (%)	8 *(72.7)*	42 *(67.7)*	
**Number of metastatic organs** ***(valid cases)***			
		
	***(11)***	***(67)***	0.95
≤ 2 organs, *N* (%)	9 *(81.8)*	54 *(80.6)*	
> 2 organs, *N* (%)	2 *(18.2)*	13 *(19.4)*	
**De novo stage IV** ***(valid cases)***			
	***(12)***	***(68)***	0.85
*N* (%)	5 (41.7)	26 (38.2)	
**Previous adjuvant therapy** ***(valid cases)***			
	***(12)***	***(68)***	0.75
Yes, *N* (%)	3 *(25)*	21 *(30.9)*	
No, *N* (%)	9 *(75)*	47 *(69.1)*	
**Previous RFS** ***(valid cases)***			
	***(3)***	***(22)***	0.18
Median (IQR)	27 (23–39)	13 (5–26)	

* De novo stage IV means that patients were diagnosed with stage IV ACC. IQR = interquartile range. N = number of patients. RFS = recurrence free survival. Statistically significant outcomes are presented in bold.

**Table 4:** Comparison between patients who had clinical benefit vs. patients who progressed at the first line of treatment.

Characteristics	Clinical Benefit N° 19	Progression N° 57	*p* value
**Gender, *(valid cases)***	*(19)*	*(57)*	1.00
Male, *N* (%)	6 *(31.6%)*	18 *(31.6%)*	
Female, *N* (%)	13 *(68.4%)*	39 *(68.4%)*	
**Age at time of palliative treatment, year** ***(valid cases)***			
		
	*(19)*	*(57)*	0.17
Median (IQR)	50 (35.5–56)	51 (38–64)	
**Hormone secretion at start of palliative treatment** ***(valid cases)***			
		
	*(19)*	*(51)*	0.36
Yes, *N* (%)	4 *(21.1)*	18 *(35.3)*	
No, *N* (%)	15 *(78.9)*	33 *(64.7)*	
**Number of metastatic organs** ***(valid cases)***			
		
	*(19)*	*(56)*	0.72
≤ 2 organs, *N* (%)	16 *(84.2)*	44 *(78.6)*	
> 2 organs, *N* (%)	3 *(15.8)*	12 *(21.4)*	
***De novo* stage IV** ***(valid cases)***			
	*(19)*	*(57)*	0.17
*N* (%)	10 (52.6)	18 (31.6)	
**Previous adjuvant therapy** ***(valid cases)***			
	*(19)*	*(57)*	0.74
Yes, *N* (%)	4 *(26.3)*	18 *(31.6)*	
No, *N* (%)	14 *(73.7)*	39 *(68.4)*	

* De novo stage IV means that patients were diagnosed with stage IV ACC. IQR = interquartile range. N = number of patients.

**Table 5:** Multivariate analysis with significant predictors of OS.

**Tumor Response ^§^**	**HR**	**CI 95%**		***p***
	0.387	0.173	0.869	0.021
TTR *	0.484	0.308	0.759	0.002

^§^ Reference category: clinical benefit *vs* progression. * HR is computed on a difference of 15 months. TTR = time in target range. HR = hazard ratio. CI = confidence interval.
